# Delayed initiation of antenatal care and associated factors in Ethiopia: a systematic review and meta-analysis

**DOI:** 10.1186/s12978-017-0412-4

**Published:** 2017-11-15

**Authors:** Gezahegn Tesfaye, Deborah Loxton, Catherine Chojenta, Agumasie Semahegn, Roger Smith

**Affiliations:** 1 Research Centre for Generational Health and Ageing, Faculty of Health and Medicine, University of Newcastle, Newcastle, Australia; 2 School of Public Health, College of Health and Medical Sciences, Haramaya University, Harar, Ethiopia; 3 Mothers and Babies Research Centre, Faculty of Health and Medicine, University of Newcastle, Newcastle, Australia

**Keywords:** Delayed antenatal care, Associated factors, Ethiopia, Systematic review, Meta-analysis

## Abstract

**Background:**

Antenatal care uptake is among the key indicators for monitoring the progress of maternal outcomes. Early initiation of antenatal care facilitates the timely management and treatment of pregnancy complications to reduce maternal deaths. In Ethiopia, antenatal care utilization is generally low, and delayed initiation of care is very common. We aimed to systematically identify and synthesize available evidence on delayed initiation of antenatal care and the associated factors in Ethiopia.

**Methods:**

Studies published in English from 1 January 2002 to 30 April 2017 were systematically searched from PubMed, Medline, EMBASE, CINAHL and other relevant sources. Two authors independently reviewed the identified studies against the eligibility criteria. The included studies were critically appraised using the Joanna Briggs-MAStARI instrument for observational studies. Meta-analysis was conducted in RevMan v5.3 for Windows using a Mantel–Haenszel random effects model. The presence of statistical heterogeneity was checked using the Cochran Q test, and its level was quantified using the I^2^ statistics. Pooled estimate of the proportion of the outcome variable was calculated. Pooled Odd Ratios with 95% CI were calculated to measure the effect sizes.

**Result:**

The pooled magnitude of delayed antenatal care in Ethiopia was 64% (95% CI: 57%, 70%). Maternal age (OR = 0.70; 95% CI: 0.53, 0.93), place of residence (OR = 0.29, 95% CI: 0.16, 0.50), maternal education (OR = 0.49; 95% CI: 0.38, 0.63), husband’s education (OR = 0.44; 95% CI: 0.23, 0.85), maternal occupation (OR = 0.75; 95% CI: 0.61, 0.93), monthly income (OR = 2.06; 95% CI: 1.23, 3.45), pregnancy intention (OR = 0.49; 95% CI: 0.40, 0.60), parity (OR = 0.46; 95% CI: 0.36, 0.58), knowledge of antenatal care (OR = 0.40; 95% CI: 0.32, 0.51), women’s autonomy (OR = 0.38; 95% CI: 0.15, 0.94), partner involvement (OR = 0.24; 95% CI: 0.07, 0.75), pregnancy complications (OR = 0.23; 95% CI: 0.06, 0.95), and means of identifying pregnancy (OR = 0.50; 95% CI: 0.36, 0.69) were significantly associated with delayed antenatal care.

**Conclusion:**

Improving female education and women’s empowerment through economic reforms, strengthening family planning programs to reduce unintended pregnancy and promoting partner involvement in pregnancy care could reduce the very high magnitude of delayed antenatal care in Ethiopia.

**Trial registration:**

CRD42017064585.

**Electronic supplementary material:**

The online version of this article (10.1186/s12978-017-0412-4) contains supplementary material, which is available to authorized users.

## Plain English summary

A professional care provided to women during pregnancy is called antenatal care. Antenatal care plays a great role in the improvement of maternal health. In Ethiopia and other sub-Saharan African countries, antenatal care utilization among pregnant women was low. Moreover, the pregnant women in Ethiopia and other developing countries tend to postpone their first antenatal care clinic visit into the later months of pregnancy. This study summarized the existing evidence on the level of late antenatal care visit and its contributing factors among pregnant women in Ethiopia. Using different databases and other sources, this review identified twenty two relevant studies that reported late antenatal care clinic visit and its influencing factors among pregnant women in Ethiopia. We summarized and analysed the reports from the twenty two studies and put a combined assessment result on the level of late antenatal care and associated factors. Based on our review, nearly two thirds of the pregnant women in Ethiopia made their first antenatal care clinic visit late in their pregnancy. Women’s non-attendance of education, husband’s non-attendance of education, women’s older age, rural dwelling, having previous births, unintended pregnancy, women’s unemployment, low monthly income, lack of knowledge about antenatal care, lack of women’s decision making power, no partner involvement, and not facing problems during pregnancy were factors associated with higher level of women’s late appearance to antenatal care. Nationwide all rounded efforts targeting the major contributing factors should be established to alleviate women’s late antenatal care utilization in the country.

## Background

The burden of maternal mortality remains hugely varied between developing and developed countries [1]. In developing countries, the overall life time risk of woman’s death due to pregnancy and related causes is estimated to be 1 in 180, while for developed countries it is about 1 in 4900 [2]. The maternal mortality ratio in Ethiopia is still high at 353 per 100,000 live births in 2015 [3], and it remains among the highest in the world. In developing countries like Ethiopia, obstetric complications during pregnancy and childbirth are the leading causes of death among reproductive aged women [3, 4]. It is generally recognized that a lack of access to, and inadequate utilization of, antenatal care (ANC) during pregnancy contributes to adverse maternal health outcomes such as maternal mortality [5, 6], something which is more common in resource-poor settings. Antenatal care uptake is one of the key indicators for monitoring the progress of improving maternal outcomes. Early initiation of ANC facilitates the timely management and treatment of pregnancy complications to reduce maternal deaths [7].

In Ethiopia, the main direct causes of maternal mortality are haemorrhage, hypertensive disorders of pregnancy, unsafe abortion and puerperal sepsis [8, 9]. These complications can be averted or otherwise treated through providing skilled care during pregnancy, child birth and in the postnatal period [2]. In 2002, the World Health Organization (WHO) recommended that pregnant women make at least four ANC visits [10]; in 2016 this recommendation was modified to at least eight visits [11], with the first ANC visit to be undertaken before the 12th week of pregnancy. While there has been marked progress in the uptake of at least one ANC attendance in Ethiopia [12–17], there has been suboptimal attendance of the recommended visits [4, 13, 18, 19]. Of even more concern was the substantial proportion of women who delayed their first ANC visit to the second or third trimester of pregnancy [5, 19–21]. According to the National Demographic and Health Survey Report of Ethiopia [20], in 2014 more than three quarters of pregnant women initiated their first visit after 16 weeks of pregnancy. Early initiation of ANC plays a paramount role in enhancing maternal health as it provides an opportunity for the early screening, treatment and referral of pregnancy complications [11]. Evidence has shown that pregnant women who initiate ANC early were less likely to develop unfavourable obstetric outcomes as compared to women who entered into care after the first trimester [22, 23].

The key challenges that women face when seeking maternal health services were clearly explained in the three delays model [24]. This model described the barriers to utilizing maternal health services at three interrelated levels before the occurrence of maternal death. At the first level, the home or community level, women may be delayed from seeking ANC due to factors such as the low social status of women in relation to decision-making, poor awareness of pregnancy or birth complications, previous poor experience of care, traditional or social practices during pregnancy or childbirth, acceptance of maternal death as normal and financial dependency. In Ethiopia, there is huge gap in the level of income among women and men especially in rural parts of Ethiopia, and women are less empowered to access and control household resources [25]. This could influence their capacity to make decisions about utilization of maternal care. Moreover, the financial burden associated transportation to and from the facility and the costs incurred for the maternal care itself profoundly diminished the uptake of the care [26]. In the second level, there may be a delay in reaching a health facility which might be due to distance, unavailability of infrastructure (road or transportation) or difficult terrain. The third level of delay (delay in receiving adequate care) might be related to a shortage of, or inadequately trained health staff, and unavailability of medical supplies and equipment.

Several studies [27–33] have investigated factors affecting delayed attendance of ANC in Ethiopia. Nonetheless, none of these studies have systematically reviewed the factors to show their overall pooled effect on delayed initiation of ANC at the national level. In addition, there were inconsistencies in attributing the influence of the factors on late initiation of ANC across various studies. For instance, there were incongruent findings on the influence of maternal education [34–37], maternal age [32–34, 36], place of residence [28, 32, 38, 39], maternal occupation [30, 34, 37, 40], marital status [32, 36, 37], husband’s education [31, 32, 41], previous experience of using ANC [32, 33, 35] and history of abortion [31, 32, 42] on delayed initiation of ANC among many other factors. Hence, demonstrating a pooled effect of the factors on delayed initiation of ANC was warranted.

Previous systematic reviews conducted in developing [43, 44] and developed [45] countries have mainly reviewed evidence on the adequacy of the utilization of ANC and its related factors. In particular, the reviews covered larger geographical regions and hence failed to reflect country specific situations. Moreover, these reviews did not centre on delayed initiation of ANC as a primary outcome of interest. The objective of this review is to systematically identify and synthesize existing evidence to understand the level of delayed initiation of ANC and associated factors among reproductive aged women in Ethiopia.

## Method

### Development of the review method

The methodology of this systematic review was developed based on the Preferred Reporting Items for Systematic Reviews and Meta-Analyses Protocols (PRISMA-P) 2015 Statement [46] and the items in the PRISMA-P checklist were addressed (Additional file 1). The four phases that were drawn from the PRISMA flow chart ( [47]) were documented in the results to show the study selection process from initially identified records to finally included studies. The protocol for this systematic review and meta-analysis was registered in international prospective register of systematic reviews (PROSPERO) and obtained the registration number (CRD42017064585).

### Search strategy

The literature search was carried out by the primary author (GT). The search was limited to papers published in English from 1 January 2002 to 30 April 2017. The year 2002 was selected, since WHO had introduced the Focused ANC model [10] by this year. We applied MeSH terms, Emtree, CINAHL headings and combined key words to identify studies in the databases. Major medical electronic databases such as PubMed, Medline (OVID interface), Excerpta Medica (Embase) (OVID interface), and CINAHL (EBSCO host) were used to identify relevant literature for the review. To cover grey literature, we hand-searched literature using the Google search engine and Google Scholar; official WHO websites; online libraries of academic and government institutions and references of electronically identified articles. The search strings or terms were stemmed from the following key words: delayed initiation, ANC, associated factors, and Ethiopia. The search terms were used to retrieve relevant literature in combined form adapted to the requirement of the specific database. Further information regarding the search strategy of the selected databases is attached (Additional file 2).

### Eligibility criteria

We included all observational studies as well as Demographic and Health Surveys (DHS) reports. We considered studies that examined the level and factors associated with delayed initiation of ANC among reproductive aged women (15–49 years) who were pregnant or gave birth at least once and who live in Ethiopia. We included studies that defined the main outcome variable “delayed initiation of ANC” as entry into care after at least 12 weeks of pregnancy, including studies that defined delayed initiation of ANC as entry into the care after 16 weeks of gestation. Studies that had been conducted in either a community or facility setting and which involved analysis of primary or secondary data were included. We included studies that had measure of association statistics or had test statistic that explicitly demonstrated the influence of the predictors on delayed initiation of ANC or had a crosstab showing the difference in magnitude of the outcome variable in the categories of the predicting variables. We excluded reviews, editorials, case series and case reports on delayed initiation of ANC. We also excluded studies that only reported qualitative findings on delayed ANC initiation. In studies that reported both quantitative and qualitative results, we only considered the quantitative findings.

### Study selection procedure

#### Screening

First studies were identified through applying the search strings and the filters in the databases as well as other relevant sources. The identified studies were exported to the citation manager (EndNote) [48] and duplicates were excluded. The two authors (GT and AS) independently screened the studies based on the information contained in the titles and abstracts according to the inclusion criteria. Based on this screening, the titles and abstracts of the studies were classified as included, excluded, and undecided. We then obtained the full texts of all the included and the undecided studies for further eligibility assessment.

#### Eligibility of studies

The two authors (GT and AS) independently reviewed the full texts of the included and undecided categories of the studies against the eligibility criteria for final inclusion. Studies that were not eligible based on the examination of the full-text were excluded and the reasons for the exclusion were described. Disagreements between the two reviewers were resolved through discussion and consensus.

#### Quality assessment

All of the included studies were critically appraised for their validity. The two authors (GT and AS) checked the methodological robustness and validity of the findings using the JBI (Joanna Briggs Institute) Meta-Analysis of Statistics Assessment and Review Instrument (MAStARI) [49]. Particular attention was given to a clear statement of the objective of the study, inclusion criteria, randomness of subject selection, identification of the study subjects, and preciseness of measurement of outcomes of interest and use of appropriate statistical analysis method, as well as documentation of sources of bias or confounding. Uncertainties were resolved by joint discussion between the reviewers. The level of agreement between the two reviewers was judged using the Cohen’s Kappa (K) coefficient statistics. To calculate “K” a two by two contingency table was constructed with “High” and “Low” categories of quality assessment provided independently by the two reviewers based on set of criteria. We obtained “K” value of (0.80), and thus the level of agreement was satisfactory. In order to minimize publication bias, we searched and included both published and unpublished literature. We obtained unpublished literatures (grey literatures) through hand-searching of online libraries of academic institutions, government organizations, and agencies in addition to using Google search engine and Google scholar. We also contacted an author to seek data that was not clearly reported in the article.

#### Data extraction process

A structured data extraction template in the form of summary table was constructed for the data abstraction. The two authors (GT and AS) systematically used the data extraction template to abstract data. The summary table contained list of items pertaining to the study characteristics to concisely present all the included studies. The specific list of items included; study year, design of the study, study setting, sample size, study subjects, data collection method, and study specific predicting factors. A quantitative data of cross-tabulation between the subject’s characteristics (predicting factors) and the outcome variable was also systematically abstracted. During the data extraction of the exposure variables, we categorized the individual classifications shown for each variable in the studies into two (exposed with the outcome and non-exposed with the outcome). The non-exposed category was considered as the reference category of the variables (*e.g for place of residence, urban was the exposed and rural was the non-exposed category*). We then put the corresponding combined numerical value to make it ready for the quantitative synthesis. During the data extraction, one of the papers (Bayou et al. 2016) reported missing and incomplete data, and the principal author of the publication was contacted to request further data via email. We received a response from the author, and were provided with the requested data. Disagreements between the two review authors were resolved by face to face discussion and reached a consensus.

#### Data synthesis and statistical analysis

The individual studies were concisely described using a summary table. The summary table particularly described the characteristics of the included studies and the main findings. We conducted the quantitative synthesis using the Cochrane community Review Manager Software (RevMan version 5.3 for windows) [50]. Summary statistics (pooled effect sizes) in Odds Ratios with 95% confidence intervals were calculated. We classified the factors that showed significant association with the outcome variable into three groups based on the three delays model, though some overlapping exist between them. Forest plots were used to graphically present the meta-analysis results. The presence of statistical heterogeneity was checked by using the Chi^2^ test (Cochran Q test) at *p*-value ≤0.05. The level of heterogeneity among the studies was quantified using the I^2^ statistics [51] where substantial heterogeneity was assumed if the I^2^ value was ≥50%. We conducted meta-analysis using Mantel–Haenszel random effects model when the studies were substantially heterogeneous (I^2^ statistic ≥50%). Pooled estimate of the magnitude of the primary outcome variable was conducted using *stats direct* (http://www.statsdirect.com) statistical software [52] using Stuart-Ord (inverse double arcsine square root) method. We hypothesized that there could be variation in the factors that lead to delayed ANC between studies that defined delayed ANC based on the WHO [10] recommendation with (≥12 weeks) and country specific recommendation [53] (≥16 weeks) due to the obvious difference in magnitude of the outcome variable. Hence, subgroup analysis was conducted based on comparison of outcomes for studies that defined delayed initiation of ANC based on (≥12 weeks) and (≥16 weeks), provided an adequate number of studies were available in the two groups. The result of the review was reported according to the PRISMA guideline for reporting [54].

## Results

### Description of the studies

**Fig. 1:**
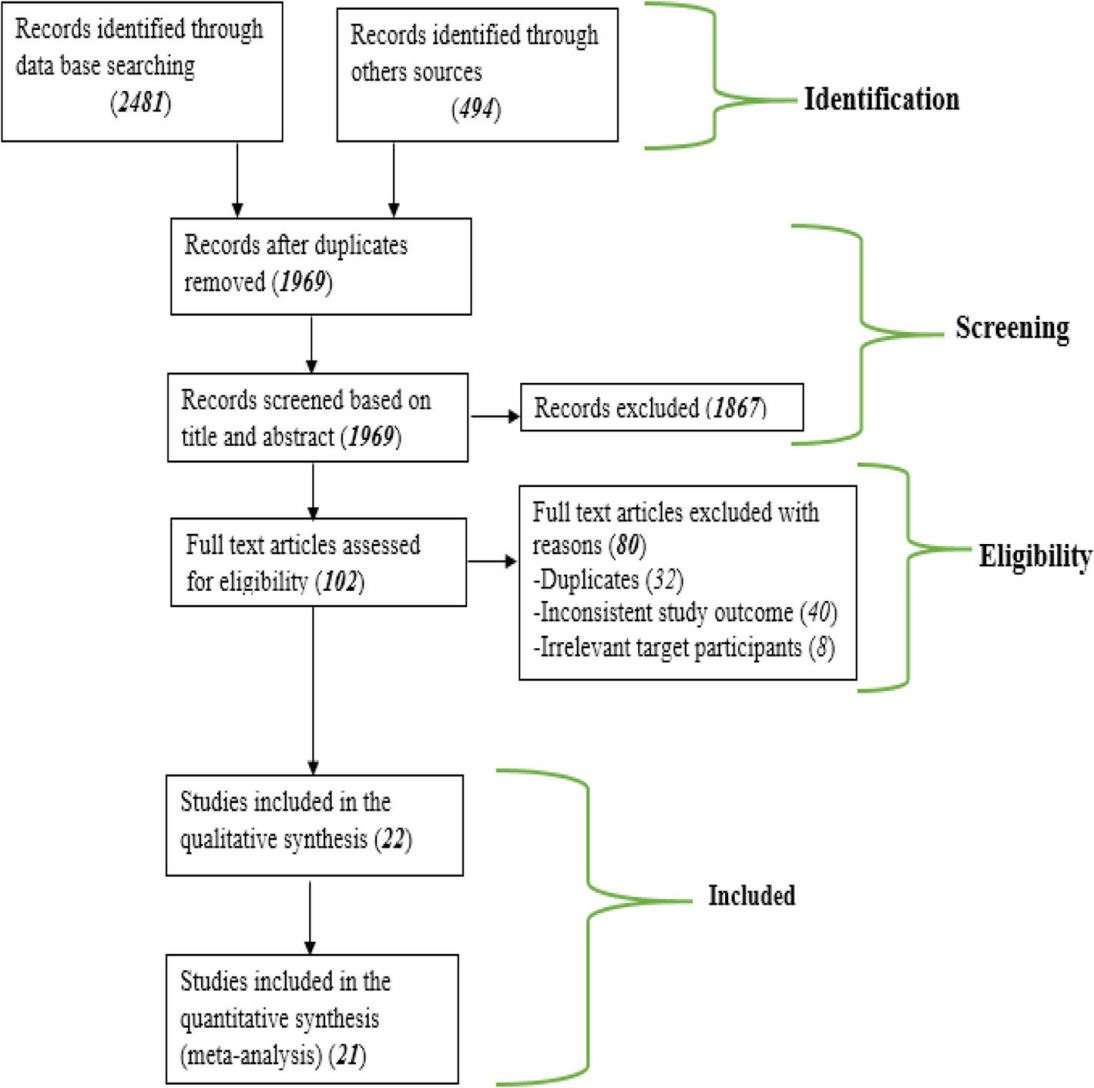
Schematic presentation of the PRISMA flow diagram to select and include studies

We retrieved 2975 studies through searching the major health and medical electronic databases and other relevant sources. From all the identified studies, 1006 articles were removed due to duplication while 1969 studies were retained for further screening. The remaining 1969 studies were then screened for their eligibility based on the title and abstract. Accordingly, 1867 studies were excluded because of the incompatibility of the content presented in the title and abstract of the studies with our review topic. Hence, the full text of the remaining 102 studies were assessed for eligibility. During the full text assessment, 80 studies were excluded from the review because of duplication, inconsistent study outcome, or irrelevant target participants. The remaining twenty two studies were critically appraised and included in the review. After the critical appraisal of the studies, we excluded one study from the quantitative synthesis due to the relatively poor methodological quality and inconsistent statistical report. Finally, twenty one studies were included for the pooled estimation of delayed initiation of ANC and factor analysis (Fig. 1). Among the included studies, there were seventeen published articles, three master theses, and one Ethiopian Demographic and Health Survey (DHS data). All of the included studies were cross-sectional by design and seventeen of the studies were conducted in a facility setting (Table 1). Ten of the studies included in the quantitative synthesis reported delayed initiation of ANC based on (≥ 12 weeks), and the remaining studies reported it based on (≥ 16 weeks).

**Table 1:** Description of the study characteristics for the included studies in the review

No	Author and year	Setting of the study	Design of the study	Sample size	Study subjects	Data collection method	Primary Outcome of Interest	Delayed ANC (definition)	Study specific predicting factors for delayed uptake of ANC
1	Amentie et al. 2015 [39]	Community based study	Cross sectional	536	Reproductive aged women who had at least one birth in the five years prior to the study	Interviewer administered questionnaire	-Utilization of ANC (uptake) -Timing of first ANC initiation	Entry in to care after 12 weeks of gestation	Place of residence (living in rural area)
2	Abosse et al. 2010 [27]	Community based study	Cross sectional	710	Reproductive aged women who had at least one birth in the five years prior to the study	Interviewer administered questionnaire	-Utilization of ANC (uptake) -Timing of first ANC visit	Entry in to care after 12 weeks of gestation	Place of residence (living in rural area)
3	Abuka et al. 2014 [35]	Facility based study	Cross sectional	406	Pregnant women attending health facility	Interviewer administered questionnaire	- Timing of first ANC booking	Entry in to care after 12 weeks of gestation	Age (≥20 year), non-attendance of formal education, high parity, perceived that timely ANC is not important, not having information, previous non-use of ANC
4	Bayou et al. 2016 [36]	Community based study	Cross sectional	814	Reproductive aged women who had at least one birth in the three years prior to the study	Interviewer administered questionnaire	-Early initiation of ANC -At least four ANC visit -Adequacy of ANC	Entry in to care after 12 weeks of gestation	Unintended pregnancy and non-attendance of formal education
5	Belayneh et al. 2014 [34]	Facility based study	Cross sectional	369	Pregnant women attending ANC service in health facility	Face-to-face interview technique	-Timing of first ANC booking	Entry in to care after 12 weeks of gestation	Non-attendance of formal education, older age [30–49], previous early ANC visit, perceived sufficient number of ANC (4+)
6	Gudayu 2015 [37]	Facility based study	Cross sectional	390	Pregnant women attending ANC service in health facilities	Face-to-face exit interview technique	-Late ANC booking	Entry in to care after 12 weeks of gestation	Not obtaining information on right time to initiate, perceived right time to book ANC (12+ weeks), non-autonomy, and use of urine test to identify pregnancy
7	Gudayu et al. 2014 [30]	Facility based study	Cross sectional	407	Pregnant women attending health facility	Face-to-face exit interview technique	-Timing of first ANC booking	Entry in to care after 12 weeks of gestation	Age (>25), younger age at marriage, pregnancy checking by means other than urine test, perceived right time to start ANC (12+ weeks), and non-autonomy
8	Yilala and Sinishaw 2015 [33]	Facility based study	Cross sectional	407	Pregnant women attending antenatal care clinic in health facility	Face-to-face exit interview technique	-Late initiation of ANC	Entry in to care after 12 weeks of gestation	Non-attendance of formal education, poor knowledge of ANC, not receiving advice from HEW, not getting advice on ANC booking, perceived right time of ANC (12+ weeks)
9	Zegeye et al. 2013 [65]	Facility based study	Cross sectional	446	Pregnant women attending health facility	Face-to-face exit interview technique	-Early ANC visit	Entry in to care after 12 weeks of gestation	High parity, lack of knowledge of ANC, unintended pregnancy
10	Tariku et al. 2010	Facility based study	Cross sectional	612	Pregnant women attending health facility	Face to face exit interview	-Timing of first ANC booking	Entry in to care after 12 weeks of gestation	High parity, unintended pregnancy, obtaining advice on when to book first ANC
11	CSA 2014 [20]	Community based study	Cross sectional (DHS data)	1571	Reproductive aged women who had at least one birth in the five years prior to the survey	Interviewer administered questionnaire	-Timing of ANC initiation -At least one ANC visit	Entry in to care after 16 weeks of gestation	Place of residence (living in rural area)
12	Damme et al. 2015 [28]	Facility based study	Cross sectional	379	Pregnant women attending ANC service in health facilities	Face-to-face exit interview technique	-Timing of first ANC booking	Entry in to care after 16 weeks of gestation	Non-attendance of formal education, rural residence, low income, having no awareness on timing of ANC
13	Ewenetu et al. 2015 [29]	Facility based study	Cross sectional	178	Pregnant women attending ANC service in health facility	Interviewer administered structured questionnaire	Late ANC initiation	Entry in to care after 16 weeks of gestation	Non-attendance of education, rural residence, no history of premature birth, late recognition of pregnancy, and unintended pregnancy
14	Fisseha et al. 2015 [66]	Facility based study	Cross sectional	410	Pregnant women attending ANC service in health facilities	Interviewer administered structured questionnaire	Timing of First ANC Booking	Entry in to care after 16 weeks of gestation	No history of still birth, no pregnancy complications, lack of knowledge of time to initiate ANC, no partner involvement on ANC
15	Gebre meskel et al. 2015 [40]	Facility based study	Cross sectional	409	Pregnant women attending ANC service in health facility	Interviewer administered structured questionnaire	Timing of First ANC Attendance	Entry in to care after 16 weeks of gestation	Low income, not receiving advice on when to start ANC, household food insecurity, unintended pregnancy
16	Girum 2016 [38]	Facility based study	Cross sectional	362	Pregnant women attending ANC service in health facilities	Face to face exit interview	Timing of First ANC Visit	Entry in to care after 16 weeks of gestation	Rural residence, low income, non-attendance of education, not receiving advice on timing of visit and unintended pregnancy
17	Gulema and Berhane 2017 [67]	Facility based study	Cross sectional	960	Pregnant women visiting health facilities for the first time	Interviewer administered structured questionnaire	Timing of First ANC Visit	Entry in to care after 16 weeks of gestation	Unemployment, low income, perceived ANC initiation time (16 weeks +), unintended pregnancy, having pregnancy complications
18	Hailesilasie and Enquselasie 2010 [41]	Facility based study	Cross sectional	419	Pregnant women attending ANC at government health facilities	Face-to-face interview of pregnant women	Late Initiation of ANC Service Utilization	Entry in to care after 16 weeks of gestation	Younger age, non-attendance of formal education, low perceived benefit of ANC, unintended pregnancy, perceived ANC initiation time (4-6 months)
19	Hussen et al. 2016 [42]	Facility based study	Cross sectional	255	Pregnant women attending ANC at government health facilities	Interviewer administered structured questionnaire	Timely Initiation of First ANC Visit	Entry in to care after 16 weeks of gestation	Non-attendance of formal education, lack of knowledge of ANC, late recognition of pregnancy, high parity
20	Lerebo et al. 2015 [31]	Facility based study	Cross sectional	415	Pregnant women attending ANC at government health facilities	Face to face interview of pregnant women	Late Booking for ANC	Entry in to care after 16 weeks of gestation	High parity, unintended pregnancy, perceived right time to book ANC (16 weeks +), no history of abortion
21	Mohammed and Berhane 2014 [68]	Facility based study	Cross sectional	383	Pregnant women attending ANC at selected public health centres	Face to face interview of pregnant women	Timing of first ANC initiation	Entry in to care after 16 weeks of gestation	Younger age, non-attendance of formal education, incorrect perception of timing of ANC, being busy
22	Tekelab and Berhanu 2014 [32]	Facility based study	Cross sectional	401	Pregnant women attending ANC service at governmental health centres	Interviewer administered structured questionnaire	Late initiation of ANC	Entry in to care after 16 weeks of gestation	Age (≥25 year), non-attendance of formal education, low monthly income, high parity, previous non-use of ANC, unintended pregnancy

From all the identified studies, 1006 were excluded during screening for duplication, and 2953 during title, abstract and full text assessment

One study was excluded due to poor methodological quality and the rest 21 studies were included in the meta-analysis

With regards to the demographic characteristics, the study participants in the included studies were pregnant women or women who have at least had one birth in the five or three years prior to the studies. The age of the participants’ were ranged from 15 to 49 years. Large majority of the participants in the included studies were urban residents. Moreover, higher proportion of the participants in the included studies were married and attended formal education (primary school and above).

### Magnitude of delayed initiation of ANC

**Fig. 2:**
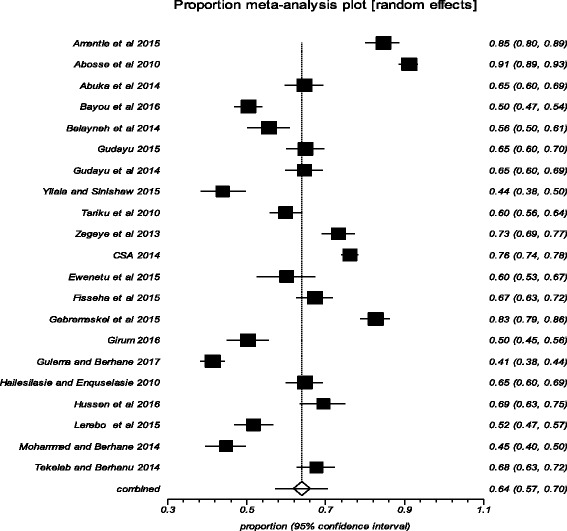
Pooled estimation of delayed initiation of ANC in Ethiopia, 2002–2017. NB (◊: Overall combined pooled proportion, and ■: Original studies proportion)

The pooled estimate of the magnitude of delayed initiation of ANC in Ethiopia was 64% (95% CI: 57%, 70%) (Fig. 2). The result of the analysis for the magnitude of delayed initiation of ANC based on the studies that reported the outcome variable with (≥ 12 weeks) was 66% (95% CI: 56%, 76%), whereas based on the studies that defined the outcome variable with (≥ 16 weeks) it was 62% (95% CI: 52%, 71%).

### Factors associated with delayed initiation of ANC

The current review revealed various factors associated with delayed initiation of ANC in Ethiopia. Significantly associated delay one factors include maternal age, maternal education, husband’s education, pregnancy intention, women’s autonomy, knowledge on ANC, partner involvement, pregnancy complication, and parity. Significantly associated delay two factors were maternal occupation, monthly income and place of residence. Means of checking pregnancy was the only delay three factor that showed statistically significant association with delayed ANC. The review also demonstrated that delay one factors such marital status and history of abortion, and delay three factor (previous use of ANC) were not significant predictors of delayed attendance of ANC services (Table 2).

**Table 2:** Overview of factors associated with delayed initiation of ANC according to the three delay model in Ethiopia, 2002–2017

Category of the factors	Significantly associated with delayed ANC (COR at 95% CI)	
	Yes	No
Delay one	Maternal age Maternal education Husband’s education Pregnancy intention Women’s autonomy Partner involvement Knowledge on ANC Presence of pregnancy complication Parity	History of abortion Marital status
Delay two	Place of residence Maternal occupation Monthly income	
Delay three	Means of checking pregnancy	Previous ANC utilization

#### Maternal age

**Fig. 3:**
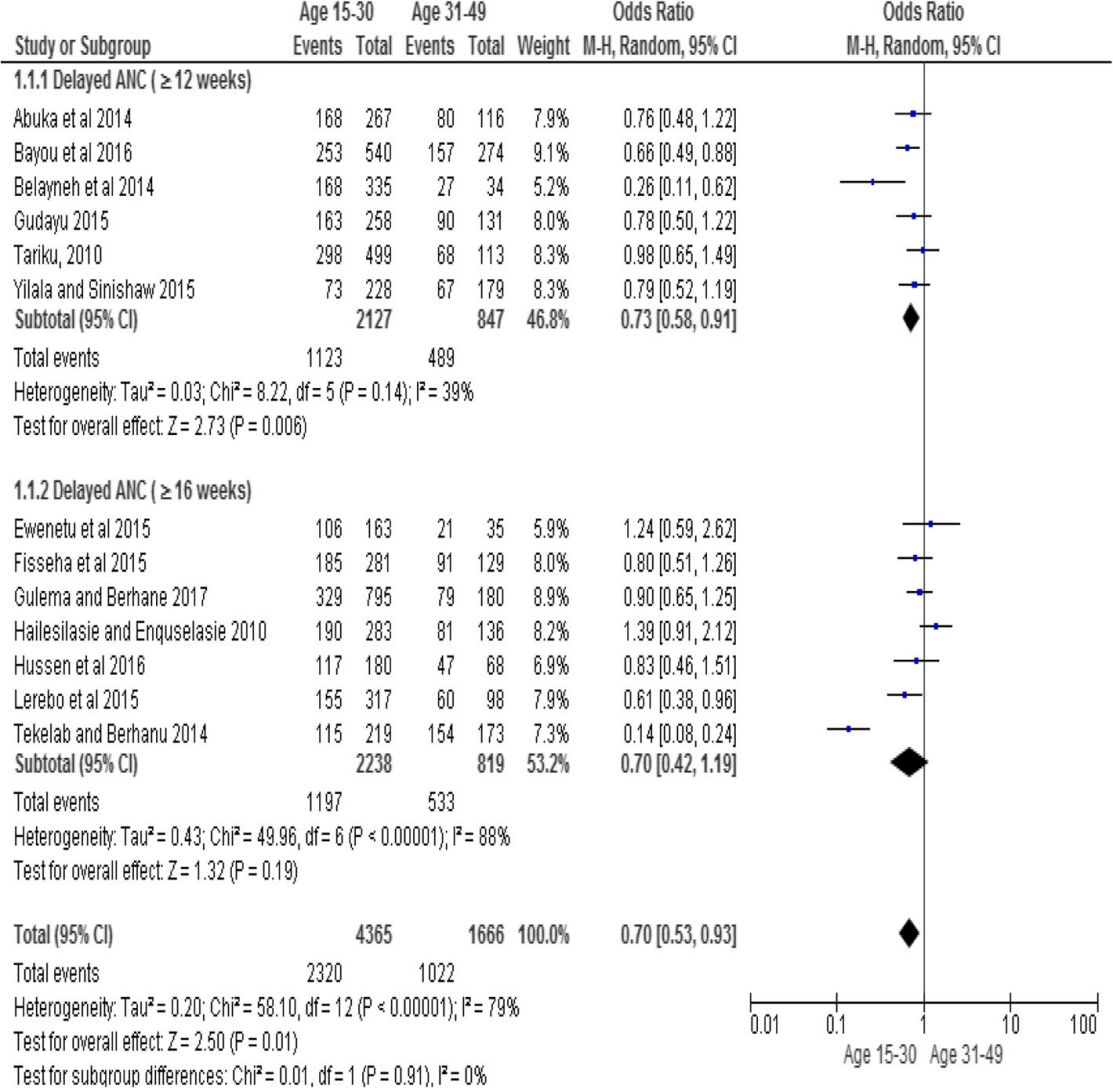
Subgroup and overall association between maternal age (reference category: age 31–49) and delayed initiation of ANC in Ethiopia, 2002–2017

Maternal age was significantly associated with delayed initiation of ANC. Women aged between 15 and 30 were less likely to have delayed their first ANC booking as compared to women aged 31 to 49 years of age (OR, 0.70; 95% CI: 0.53, 0.93). However, the subgroup (delayed initiation ≥16 weeks) showed no association between maternal age and delayed booking of ANC (OR, 0.70; 95% CI: 0.42, 1.19). But it did not affect the overall association. Random effect model was employed for the analysis as the I^2^ value was >50% (Fig. 3).

#### Maternal education

**Fig. 4:**
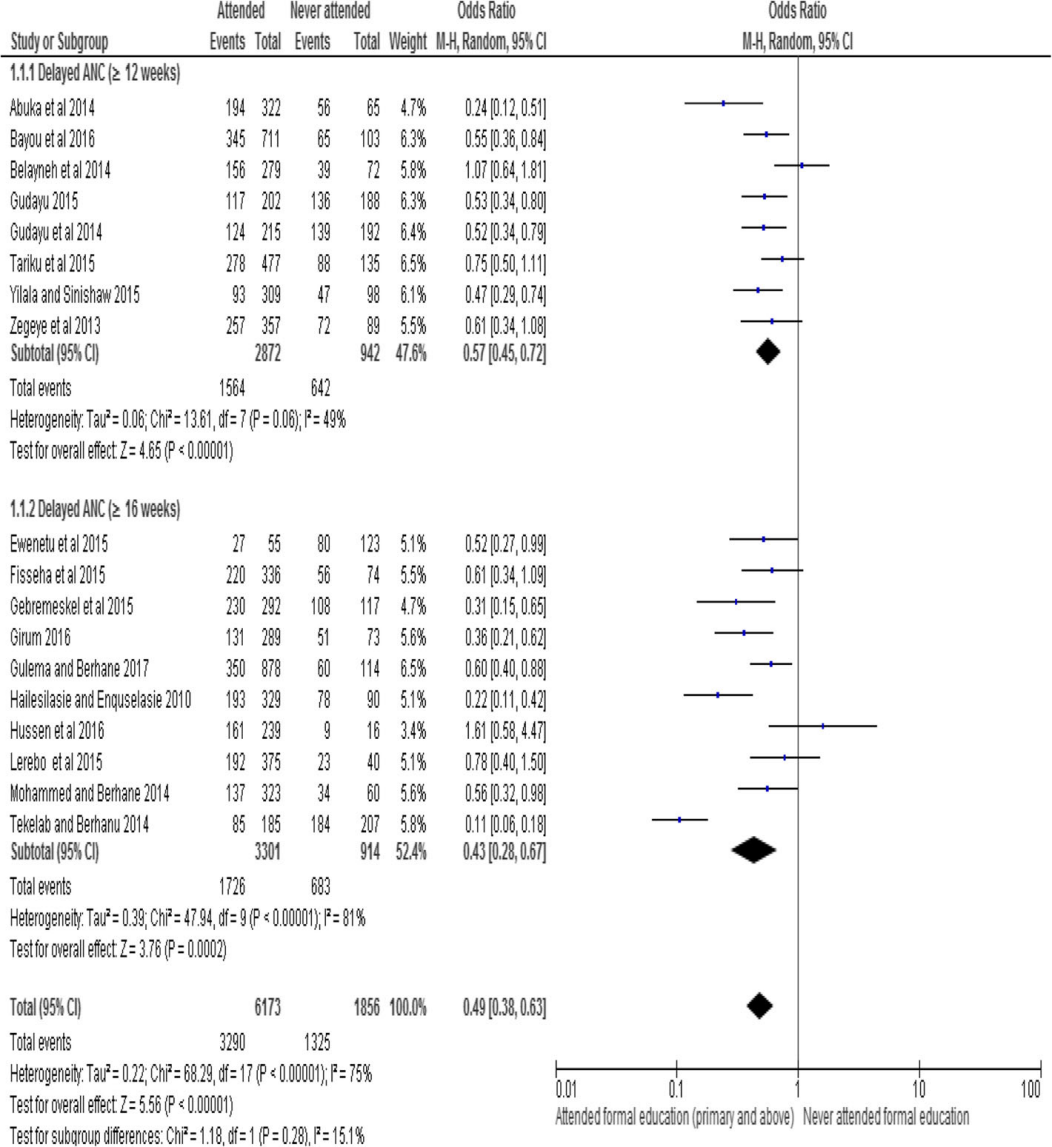
Subgroup and overall association between maternal education (reference category: never attended formal education) and delayed initiation of ANC in Ethiopia, 2002–2017

The meta-analysis showed that maternal education was significantly associated with delayed ANC initiation. The overall Odds Ratio 0.49 at 95% CI: 0.38, 0.63 indicated that women who have attended primary or above level of education were less likely to delay their first ANC visit as compared to women without formal education. In spite of the heterogeneity of the studies, the finding showed statistically significant association. The subgroup analysis for studies with (≥12 weeks) (OR, 0.57; 95% CI: 0.45, 0.72) and studies (≥16 weeks) (OR, 0.43; 95% CI: 0.28, 0.67) both showed significant association between the maternal educational status and delayed initiation of ANC. We used random effect model for the analysis since the I^2^ value was 75% (Fig. 4).

#### Place of residence

**Fig. 5:**
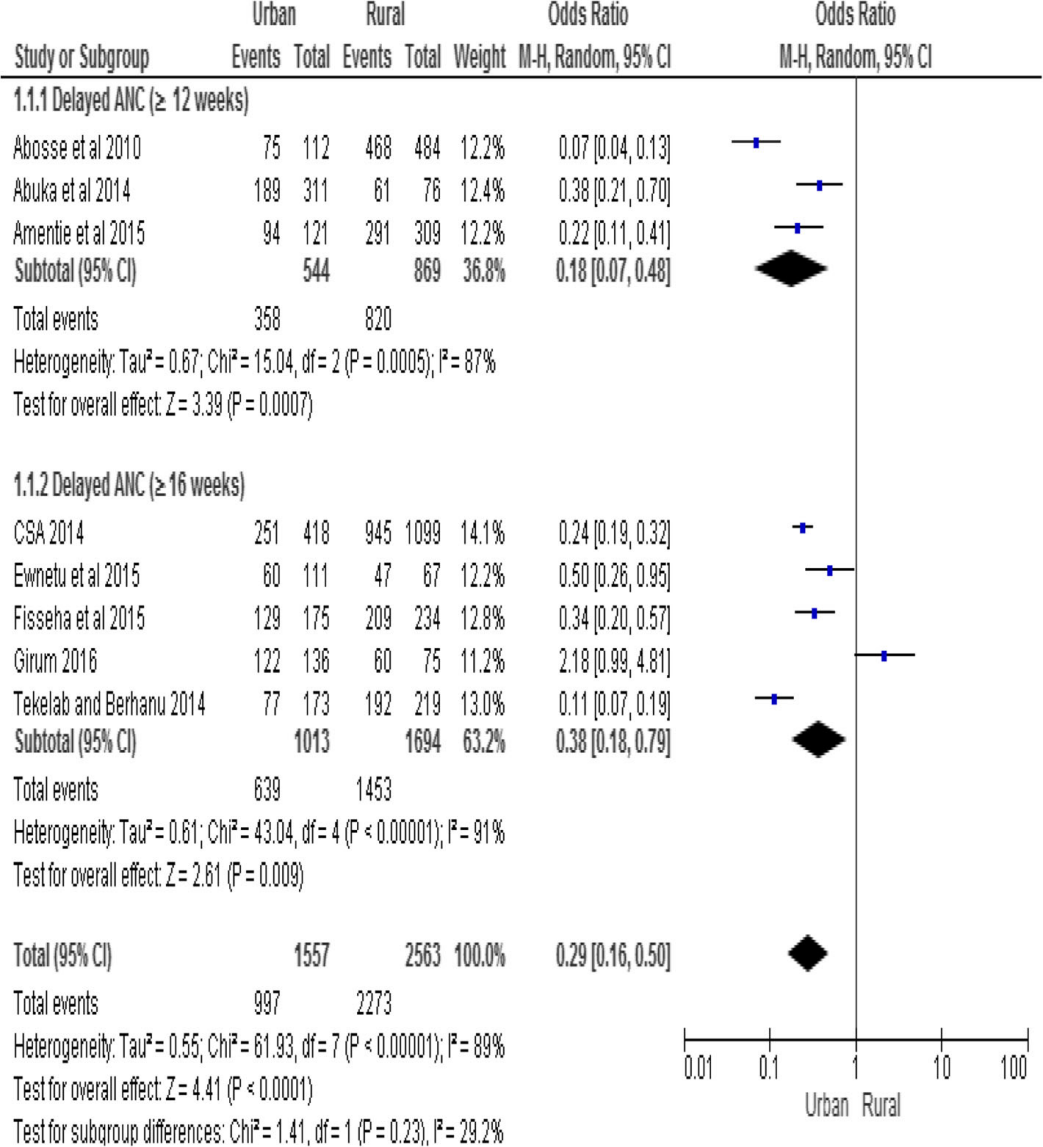
Subgroup and overall association between place of residence (reference category: rural) and delayed initiation of ANC in Ethiopia, 2002–2017

According to the factor analysis of the included studies, place of residence was significantly associated with delayed initiation of ANC. Women who live in urban area were less likely to have delayed initiation of ANC (OR, 0.29, 95% CI: 0.16, 0.50). No difference was found in terms of the direction of association between place of residence and delayed initiation of ANC in the subgroups analysis. Random effect model was used for the analysis since the heterogeneity test showed an overall I^2^ value of 89% (Fig. 5).

#### Pregnancy intention

**Fig. 6:**
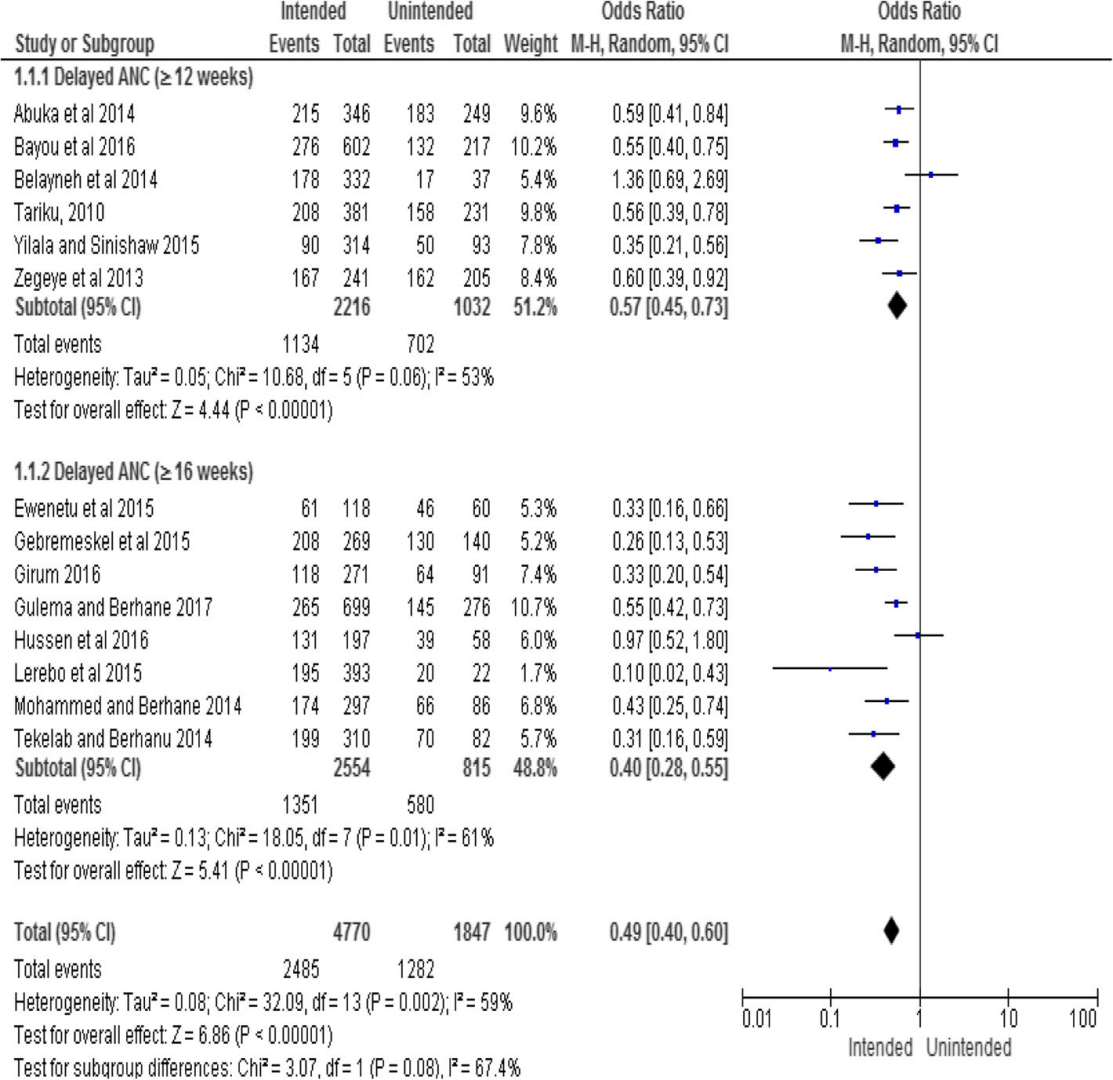
Subgroup and overall association between pregnancy intention (reference category: unintended) and delayed initiation of ANC in Ethiopia, 2002–2017

The review finding showed that women with intended pregnancy were less likely to delay their ANC initiation (OR, 0.49; 95% CI: 0.40, 0.60). There was no difference between the subgroups in the direction of association. As the heterogeneity test indicated an I^2^ value of 59%, random effect was considered for the analysis (Fig. 6).

Below are the descriptions of other factors that are associated with delayed initiation of ANC with the meta-analysis test statistics (Table 3).

**Table 3:** Summary of the test statistics of association between the remaining delay one, two and three factors with delayed initiation of ANC in Ethiopia, 2002–2017

Predictor variable	Subgroup OR (95%CI)		I^2^	Combined OR (95% CI)	Overall I^2^
Monthly income [≤1000ETB(50USD)]	I	1.77(1.16, 2.72)	65%	2.06(1.23, 3.45)	91%
	II	2.26(0.96, 5.29)	94%		
Marital status [In marriage]	I	0.92(0.55, 1.54)	77%	0.81(0.56, 1.16)	68%
	II	0.68(0.38, 1.22)	57%		
Maternal occupation [Employed]	I	0.76(0.53, 1.09)	83%	0.75(0.61, 0.93)	74%
	II	0.74(0.57, 0.97)	66%		
Husband education [Attended primary/above]	II	0.44(0.23, 0.85)	80%		
Women’s autonomy [Autonomous]	I	0.38(0.15, 0.94)	89%		
Previous use of ANC [Previous use]	I	0.65(0.42, 1.02)	45%	0.62(0.34, 1.11)	85%
	II	0.53(0.17, 1.67)	92%		
Parity [Nulliparity]	I	0.51(0.42, 0.61)	0%	0.46(0.36, 0.58)	67%
	II	0.42(0.27, 0.66)	81%		
Partner involvement [Involved]	I	0.44(0.21, 0.91)	85%	0.24(0.07, 0.75)	85%
	II	0.14(0.08, 0.22)	85%		
Knowledge of ANC [Knowledgeable]	I	0.32(0.22, 0.46)	4%	0.40(0.32, 0.51)	29%
	II	0.46(0.35, 0.62)	54%		
History of abortion [Have history of abortion]	I	1.19(0.85, 1.66)	0%	1.16(0.79, 1.69)	77%
	II	1.14(0.67, 1.95)	77%		
Pregnancy complication [Presence of complication]	II	0.23(0.06, 0.95)	97%		
Means of identifying pregnancy [Urine]	I	0.50(0.36, 0.69)	67%		

Subgroup: I = Studies that defined delayed initiation of ANC (after 12 weeks of gestation), Subgroup: II = Studies that defined delayed initiation of ANC (after 16 weeks of gestation), I^2^ is the percentage of total variance due to between study heterogeneity

#### Family monthly income

Monthly average family income was significantly associated with delayed ANC initiation. It was demonstrated that there was increased odds of delayed initiation of ANC among women with an average family income of ≤1000 ETB (50USD) compared to those women whose average family income was >1000ETB (50USD) (OR, 2.06; 95% CI: 1.23, 3.45). The association between family monthly income and delayed initiation of antennal care was not consistent across the two subgroups, where the subgroup which defined the outcome variable based on (≥16 weeks) showed insignificant association between monthly income and delayed initiation of ANC (OR, 2.26; 95% CI: 0.96, 5.29), whereas the subgroup (delayed ANC ≥12 weeks) (OR, 1.77; 95% CI: 1.16, 2.72) showed significant association. Due to the heterogeneity of the studies (I^2^ = 91%), we used random effect model for the analysis.

#### Marital status

Our systematic review demonstrated that there was no significant association between marital status and delayed initiation of ANC (OR, 0.81; 95% CI: 0.56, 1.16). The same was true in the subgroup analysis which showed no association between marital status and delayed initiation of ANC. We assumed random effect model for the analysis since the I^2^ statistics showed presence of heterogeneity (68%).

#### Maternal occupation

The overall Odds Ratio showed that there was significant association between maternal occupation and delayed initiation of ANC (OR, 0.75; 95% CI: 0.61, 0.93). Employed women were less likely to delay their ANC as compared to their counterparts. However, the subgroup (delayed ANC initiation ≥12 weeks) (OR, 0.76; 95% CI: 0.53, 1.09) showed no association between maternal occupation and delayed booking of ANC. But the overall association was not altered. The heterogeneity test showed an I^2^ value of 74%, and therefore random effect model was considered for the analysis.

#### Husband’s education

Association between husband’s education and delayed initiation of ANC was carried out in one group of studies that defined the outcome variable with (*≥* 16 weeks), as this variable was not reported in the other group of studies. The analysis showed that women having a husband who attended formal education were less likely to delay their first antenatal visit as compared to those women whose husband had never attended formal education (OR, 0.44; 95% CI: 0.23, 0.85). Random effect model was implemented for the analysis since the I^2^ value was greater than 50%.

#### Women’s autonomy

We conducted analysis of a single group of studies that defined the outcome variable with (≥12 weeks) due to the unavailability of corresponding data about women’s autonomy in the other group of studies. Accordingly, the analysis result revealed that women’s autonomy has a significant association with delayed initiation of ANC (OR, 0.38; 95% CI: 0.15, 0.94). Autonomous women were less likely to initiate their first ANC later than non-autonomous women. Random effect model was used for the analysis as the I^2^ test result is 89%.

#### Previous use of ANC

The finding revealed that there was no significant association between previous utilization of ANC and delayed initiation of ANC (OR, 0.62; 95% CI: 0.34, 1.11). This was the case in both subgroups of the studies, and the overall analysis result. We assumed a random effect model for the analysis as the I^2^ value 85% showed substantial heterogeneity between the studies.

#### Parity

Parity was another predicting factor that affected delayed initiation of ANC. In this regard, women with no parity (nulliparous) were less likely to have delayed their ANC initiation as compared to women who were primipara and above. This was demonstrated in the overall Odds Ratio, 0.46 at 95% CI: 0.36, 0.58. There was no difference in the association between parity and delayed initiation of ANC in the subgroup analysis. Since the I^2^ value was 67%, indicating considerable heterogeneity of the included studies, we assumed a random effect model for the analysis.

#### Partner involvement

We conducted the analysis using studies from both subgroups and it was found that partner involvement has a significant association with delayed initiation of ANC. Women who had a partner who was involved in ANC were less likely to delay thier first ANC initiation compared with women with no partner involvement in ANC (OR, 0.24; 95% CI: 0.07, 0.75). We considered a random effect model for the analysis because the I^2^ value was 85%.

#### Knowledge of ANC

The overall analysis of both groups of studies showed that knowledge of ANC has association with delayed initiation of ANC. Knowledgeable women were less likely to delay their ANC booking as compared to non-knowledgeable women (OR, 0.40; 95% CI: 0.32, 0.51). Fixed effect model was assumed for the analysis as the Chi square test (7.08) with the *p*-value (0.21) showed statistically insignificant heterogeneity among the included studies for this factor analysis.

#### History of abortion

We found no significant association between history of abortion and delayed initiation of ANC (OR, 1.16; 95% CI: 0.79, 1.69), and this was true in the analysis result of both subgroups of studies. We assumed a random effect model since the I^2^ statistics (77%) showed substantial heterogeneity.

### Pregnancy complications

There was significant association between the presence of complications during pregnancy and delayed initiation of ANC on a single group analysis (delayed initiation of ANC ≥ 16 weeks). Women who experienced complications during pregnancy were less likely to delay their first ANC attendance compared to women who did not experience complications during pregnancy (OR, 0.23; 95% CI: 0.06, 0.95). Random effect model was used for the analysis since the I^2^ value was greater than 50%.

#### Means of identifying pregnancy

No sub-group analysis was performed due to lack of relevant statistics with regards to means of identifying pregnancy in one group (delayed initiation of ANC ≥ 16 weeks). Single group (delayed initiation of ANC ≥ 12 weeks) analysis however showed a significant association between means of identifying pregnancy with delayed initiation of ANC. Women who identified their pregnancy with a urine test were less likely to delay their first ANC visit as compared to women who identified their pregnancy using other means (OR, 0.50; 95% CI: 0.36, 0.69). We considered random effect model for the analysis since the I^2^ value was greater than 50%.

## Discussion

Maternal age, maternal education, husband’s education, maternal occupation, place of residence, parity, knowledge of ANC, women’s autonomy, partner involvement, pregnancy intention, presence of pregnancy complications, and means of identifying pregnancy were significantly associated factors for delayed initiation of ANC in Ethiopia. We found out that nearly two thirds of the women in Ethiopia initiated their first ANC late after 12^th^ week of pregnancy. Marital status, history of abortion and previous use of ANC showed no significant association with delayed initiation of ANC.

Timely initiation and continuous attendance of ANC is believed to improve maternal health outcomes [23, 55]. This is the case particularly in developing countries where the health status of women is very poor. It is imperative to understand the overall level of delayed initiation of ANC and the contributing factors at the country level to inform current efforts to improve maternal outcomes through adequate utilization of ANC in Ethiopia. The current systematic review supplied a summary of available evidence on the level of delayed initiation of ANC and associated factors in Ethiopia. The importance of systematic reviews to provide relevant information to transform health care delivery system and policy modification or ratification was well documented [56]. This systematic review summarized up-to-date empirical evidence and fleshed out key areas of action regarding delayed initiation of ANC in Ethiopia. This is an important step-forward to ensure maternal health program planners and policy makers in the country make informed decisions regarding where the corrective measures should be instituted and maximized.

Even though the WHO [10, 11] recommended initiation of ANC attendance not later than the first trimester of pregnancy, the reviewed evidence showed that the magnitude of delayed initiation of ANC is very high, at 64% in Ethiopia. This figure was almost in line with a comparative report of demographic and health survey data of twenty one sub-Saharan African countries [21] where, on average, more than two-thirds of the reproductive aged women initiated their first ANC after the first trimester of pregnancy. This might be due to several socio-cultural, economic and contextual factors including women’s poor decision making power at a household level due to deeply rooted gender inequality, poor educational status, and poverty, which in turn could limit the women’s ability to seek care earlier. The decision to early seek care and assistance during pregnancy among Ethiopian women especially in rural areas are linked with many cultural practices [57, 58], which were barrier to accessing services throughout a woman’s pregnancy. Delayed initiation of ANC was a significant risk factor for maternal death, particularly among the disadvantaged women [22]. Hence countries need to prioritise efforts to improve the initiation of ANC.

According to this review, maternal age and education, husband’s education, parity, knowledge of ANC and women’s autonomy were influencing factors for delayed first ANC attendance in Ethiopia. The result of the current review was in agreement with the systematic review of studies [43, 44, 59] conducted in other settings where maternal age, maternal education, husband’s education, and parity were the influencing factors for delayed initiation of ANC. The possible reason for older women aged 31 to 49 delaying their first ANC might be that they most likely are uneducated, have poor knowledge of ANC, have experienced pregnancies without complications previously, are less fearful unlike younger women and may be more likely to be multiparous. Education of the mother and husband could play a great role in improving awareness of health matters in general, and the importance of ANC in particular. Having a better awareness may enable women to seek ANC and utilize the service early in pregnancy. This was particularly reflected in the systematic review of studies among non-western women in industrialized countries [45] where women’s low level of educational status was associated with late entry into ANC.

Furthermore, a lack of knowledge about ANC is positively associated with delayed initiation of ANC. Women who had been provided with information regarding ANC, pregnancy risks and danger signs were more likely to initiate ANC early compared to women who did not have knowledge of these issues. This could motivate the women to initiate ANC early to better avoid the risks associated with pregnancy. It is anticipated that well informed women were more likely to make judicious choices about the proper utilization of ANC. It was also found that women’s autonomy was a significant predictor of delayed initiation of ANC where non-autonomous women were more likely to postpone ANC, which could be due to the fact that they were under the influence of their partner or family (especially in male headed households), restricted to comply with family norms, had lack of family or social support, and a partner who was not available or who refused to accompany them. This was demonstrated in a systematic review of studies in the developing countries [44], where social support from family members, extent of ties within social networks, and obtaining health information from these sources highly influence timely utilization of ANC.

Additionally, the meta-analysis revealed that place of residence, maternal occupation, monthly income, and partner involvement were significantly associated with delayed ANC initiation. Rural women were more likely to delay their first ANC attendance than urban women. This could be explained by the fact that urban women would most likely have easy access to health care facilities, have a good awareness of health matters, and have better exposure to media. Moreover, unemployed women were more likely to delay initiation of ANC as compared to employed women. A similar finding was reported in other systematic reviews [43, 45] where not being in employment explained women’s delayed entry into the care. It was also evidenced that women with high economic status were more likely to receive ANC earlier than those with a lower economic status [43, 44]. These financial constraints are in turn related to other barriers to seeking help, including transportation costs, the cost of obtaining care, or laboratory tests [60, 61].

Moreover, our finding suggests that women whose partner was involved in ANC were less likely to delay their first ANC attendance than women whose partner was not involved in ANC. Partner involvement in terms of initiating and/or supporting the idea to utilize ANC early, or by accompanying the pregnant mother to the health facility may have an important impact on the early attendance of ANC. In many traditions, the involvement of men in reproductive health has not been considered an important issue. In general male partners did not accompany their wives to attend ANC and other maternal health services [43]. The husband’s lack of involvement in ANC may immensely affect the women’s capability to initiate ANC early. It was found in a systematic review [62] that the involvement of men in ANC has a positive influence on the overall uptake of the service and its early attendance.

Furthermore, the meta-analysis identified factors such as pregnancy intention, presence of pregnancy complications and means of identifying pregnancy as an important factors that affect delayed initiation of ANC. This finding is consistent with a systematic review of small scale studies conducted in both developed and developing countries [63] on the relationship between pregnancy intention and timely initiation as well as obtaining adequate ANC. It was revealed that unintended pregnancy has a strong association with delayed initiation of first ANC services. Another systematic review [44] confirmed that women whose pregnancy was unintended tended to initiate ANC later than the first trimester of pregnancy. With regards to complications during pregnancy, the findings of this study is similar to a systematic review of literature [43] conducted in developing countries where pregnant women who did not experience obstetric complications were more likely to delay their first ANC compared to their counterparts.

In the current systematic review and meta-analysis, we observed some discrepancies in the included studies in defining the outcome variable “delayed initiation of ANC”. Half of the included studies defined delayed initiation of ANC based on the cut-off point of 12 weeks of gestation, whereas the rest of the studies defined it based on 16 weeks. However the WHO [10, 11] defined late ANC initiation as entry into care after 12^th^ week of pregnancy. Conversely, in this review we noticed a contrasting type of definition across several studies [27–42] as well as ANC practice in health facilities [53], implying that there was poor compliance of the WHO recommendation on the timing of first ANC initiation in Ethiopia. Countries might prefer to adapt or contextualise the original clinical practice guidelines with some changes, depending on their setting, to effectively implement the recommendations. Even if recommendations from the parent clinical practice guidelines can be adapted, how they are implemented needs to address local issues. Thus countries may need to contextualise guideline by addressing those implementation issues so that care becomes more relevant to the local environments [64]. However, at least there should not be inconsistencies between the implemented specific health recommendation within the country’s health care delivery system and the health research arena. Hence, we recommend to concerned parties in the health sector in Ethiopia, particularly the health research scholars, that there is a need to adhere to the WHO recommended guideline on the timing of ANC initiation. Moreover, any further adapted or contextualised guideline on the timing of ANC initiation needs to be followed or implemented consistently in a standardized way.

The current systematic review and meta-analysis was not without limitations. The first limitation was the exclusion of qualitative studies from the review, which might reveal other important factors affecting women’s behaviour to delay ANC attendance or might otherwise corroborate the quantitative findings. Secondly, since our meta-analysis used Crude Odds Ratios, it might be difficult to fully ascertain the effect of the exposure factors on the outcome of interest. Thirdly, as all the included studies were cross-sectional by design, it is difficult to establish temporal relationship between the outcome and exposure variables. Lastly, conducting meta-analysis despite the inherent heterogeneity between the included studies might have affected the quantitative findings. Our systematic review and meta-analysis also has some strengths. In this regard, we considered selection and inclusion of both published and unpublished literature which has the potential to minimize publication bias. Moreover, our search strategy was extensive using a number of major medical databases and other search engines. Lastly, we conducted a sub-group analysis of studies that employed different definitions of delayed initiation of ANC to appreciate the independent subgroup findings.

## Conclusion

The current review revealed that nearly two thirds of women were delaying their first ANC visit in Ethiopia. The review pointed out various factors attributed to high level of delayed initiation of ANC in Ethiopia. Among these maternal age, place of residence, maternal education, husband’s education, maternal occupation, family monthly income, pregnancy intention, parity, knowledge of ANC, women’s autonomy, partner involvement, problem during pregnancy, and means of identifying pregnancy showed significant association with delayed initiation of ANC. Therefore, intervention efforts to improve ANC utilization in Ethiopia require targeting these impeding factors. Moreover, strategies should be designed to intensify advocacy of female education, women’s empowerment activities need to be continued through economic reforms, family planning programs should be strengthened to reduce unintended pregnancies, and partner involvement in ANC should be promoted through different means of communication. Further qualitative studies are recommended to gain further insight into the societal and health system barriers that contribute to delayed initiation of ANC in Ethiopia.

## Additional files

Additional file 1: PRISMA-P (Preferred Reporting Items for Systematic review and Meta-Analysis Protocols) 2015 checklist: recommended items to address in a systematic review protocol*. (DOC 85 kb)

Additional file 2: Search Strategy. (DOCX 28 kb)

## Abbreviations

ANC: Antenatal care
CI: Confidence interval
DHS: Demographic and Health Survey
ETB: Ethiopian birr
JBI: Joanna Briggs Institute
MAStARI: Meta-Analysis of Statistics Assessment and Review Instrument
M-H: Mantel–Haenszel
NSW: New South Wales
OR: Odds ratio
PRISMA: Preferred Reporting Items for Systematic Reviews and Meta-Analyses Protocols
USD: United States Dollar
WHO: World Health Organization
