# Supplementary material for: Delayed initiation of antenatal care and associated factors in Ethiopia: a systematic review and meta-analysis
Source: Reprod Health. 2017 Nov 15;14:150. doi: 10.1186/s12978-017-0412-4 (PMC5688656; doi:10.1186/s12978-017-0412-4)
Supplement: Supplementary file 2 — Search Strategy. (DOCX 28 kb) [file 12978_2017_412_MOESM2_ESM.docx]

Additional file 2: **Search Strategy.**

**Title of the review:** Delayed initiation of antenatal care and associated factors in Ethiopia.

| **No** | **Database** | **Search filters (Year=01/01/2002 to 30/04/2017, Language=English), DATE OF THIS SEARCH [MAY 05, 2017]** | |
| --- | --- | --- | --- |
|  |  | **Combined search phrase** | **Search results** |
| 1 | **PubMed** | (((((((((("Delayed initiation"[All Fields] OR "Late initiation"[All Fields]) OR "Delayed utilization"[All Fields] OR "late utilization"[All Fields] OR Delayed[All Fields] OR late[Title/Abstract] OR initiation[Title/Abstract] OR utilization[Title/Abstract] OR "Delayed attendance"[All Fields] OR "Attendance"[Title/Abstract]) OR "Late booking"[All Fields] OR "Booking"[Title/Abstract]) AND ((((((((("Antenatal care"[All Fields] OR "First antenatal care"[All Fields] OR "Prenatal care"[Mesh]) OR "Antepartum care"[All Fields]) OR "ANC"[All Fields]) OR "PNC"[All Fields]) OR "Antenatal follow up"[All fields]) OR "Antenatal period"[All Fields]) OR "Antenatal service"[All Fields]) OR "Antenatal care visit"[All Fields]) OR "Antenatal presentation"[All Fields]) OR "Antenatal attendance"[All Fields]) OR "Perinatal care"[Mesh]) OR "Maternal health services"[Mesh]) AND (((("Associated factors"[All Fields] OR "Factors associated"[All Fields]) OR "Influencing factors"[All Fields]) OR "Predictors"[All Fields]) OR "Predicting factors"[All Fields]) OR "Correlates"[All Fields]) OR Determinants[All Fields]) OR "Risk factors"[Mesh]) AND "Ethiopia"[Mesh]) AND (("2002/01/01"[PDAT] : "2017/04/30"[PDAT]) AND English[lang]) | **1011** |
| 2 | **Medline** | \| **#** \| **Searches** \| **Results** \| \| --- \| --- \| --- \| \| 1 \| 'Delayed initiation'.mp. \| 590 \| \| 2 \| exp Pregnancy/ or 'Late initiation'.mp. or exp Prenatal Care/ \| 833510 \| \| 3 \| 'Delayed utilization'.mp. \| 13 \| \| 4 \| *Health Services/ or 'late utilization'.mp. or exp Prenatal Care/ \| 39335 \| \| 5 \| Delayed.mp. \| 308828 \| \| 6 \| late.mp. or exp Pregnancy/ \| 1169933 \| \| 7 \| initiation.mp. \| 214560 \| \| 8 \| utilization.mp. \| 174823 \| \| 9 \| exp "Patient Acceptance of Health Care"/ or 'Delayed attendance'.mp. \| 209087 \| \| 10 \| Attendance.mp. or exp Patient Compliance/ \| 85993 \| \| 11 \| exp Pregnancy Complications/ or exp Pregnancy Outcome/ or exp Pregnancy/ or exp Prenatal Care/ or 'Late booking'.mp. or exp Gestational Age/ \| 882197 \| \| 12 \| *Obstetric Labor Complications/ or exp Pregnancy/ or exp Prenatal Care/ or exp Pregnancy Complications/ or 'Antenatal care'.mp. or exp Pregnancy Outcome/ or exp Maternal Health Services/ \| 867354 \| \| 13 \| exp Pregnancy/ or exp Prenatal Care/ or 'First antenatal care'.mp. or exp Pregnancy Complications/ \| 860196 \| \| 14 \| exp Pregnancy/ or exp Prenatal Care/ or exp Pregnancy Complications/ or 'Prenatal care'.mp. or exp Maternal Health Services/ \| 867282 \| \| 15 \| exp Pregnancy Complications/ or exp Pregnancy/ or 'Antepartum care'.mp. or exp Maternal Health Services/ or exp Prenatal Care/ \| 865783 \| \| 16 \| exp Pregnancy/ or ANC.mp. or exp Prenatal Care/ \| 835885 \| \| 17 \| exp Pregnancy/ or PNC.mp. or exp Prenatal Care/ \| 834055 \| \| 18 \| exp Pregnancy Outcome/ or exp Pregnancy Complications/ or exp Pregnancy/ or *Obstetric Labor, Premature/ or 'Antenatal follow up'.mp. or exp Prenatal Care/ \| 860180 \| \| 19 \| exp Prenatal Care/ or exp Pregnancy/ or 'Antenatal period'.mp. or *Obstetric Labor Complications/ or exp Pregnancy Complications/ \| 860377 \| \| 20 \| exp Pregnancy/ or exp Prenatal Care/ or exp Pregnancy Complications/ or 'Antenatal service'.mp. or exp Maternal Health Services/ \| 865783 \| \| 21 \| exp Pregnancy/ or exp Prenatal Care/ or 'Antenatal care visit'.mp. or exp Maternal Health Services/ \| 839235 \| \| 22 \| exp Pregnancy/ or 'Antenatal presentation'.mp. \| 831456 \| \| 23 \| exp "Patient Acceptance of Health Care"/ or exp Pregnancy/ or exp Prenatal Care/ or exp Maternal Health Services/ or exp Pregnancy Complications/ or 'Antenatal attendance'.mp. \| 1064368 \| \| 24 \| exp Perinatal Care/ or 'Perinatal care'.mp. \| 9889 \| \| 25 \| *Delivery, Obstetric/ or exp Pregnancy/ or exp Maternal Health Services/ or exp Prenatal Care/ or 'Maternal health services'.mp. or *Maternal Welfare/ \| 841762 \| \| 26 \| exp Cross-Sectional Studies/ or 'Associated factors'.mp. \| 255598 \| \| 27 \| exp Risk Factors/ or 'Factors associated'.mp. \| 761229 \| \| 28 \| 'Influencing factors'.mp. \| 5740 \| \| 29 \| Predictors.mp. \| 181574 \| \| 30 \| 'Predicting factors'.mp. \| 918 \| \| 31 \| Correlates.mp. \| 144337 \| \| 32 \| Determinants.mp. or exp "Social Determinants of Health"/ \| 133112 \| \| 33 \| exp Risk Factors/ or 'Risk factors'.mp. \| 864224 \| \| 34 \| Ethiopia.mp. or exp Ethiopia/ \| 12131 \| \| 35 \| exp Pregnancy/ or exp Pregnancy Complications/ or booking.mp. or exp Prenatal Care/ \| 860766 \| \| 36 \| 1 or 2 or 3 or 4 or 5 or 6 or 7 or 8 or 9 or 10 or 11 or 35 \| 2050883 \| \| 37 \| 12 or 13 or 14 or 15 or 16 or 17 or 18 or 19 or 20 or 21 or 22 or 23 or 24 or 25 \| 1073570 \| \| 38 \| 26 or 27 or 28 or 29 or 30 or 31 or 32 or 33 \| 1463433 \| \| 39 \| 34 and 36 and 37 and 38 \| 668 \| \| 40 \| limit 39 to (english language and yr="2002 -2017") \| 611 \| | **611** |
| 3 | **EMBASE** | \| **#** \| **Searches** \| **Results** \| \| --- \| --- \| --- \| \| 1 \| exp therapy delay/ or 'Delayed initiation'.mp. \| 9693 \| \| 2 \| exp prenatal care/ or 'Late initiation'.mp. \| 132377 \| \| 3 \| exp health care utilization/ or 'Delayed utilization'.mp. \| 53553 \| \| 4 \| 'late utilization'.mp. or exp pregnancy/ \| 705334 \| \| 5 \| Delayed.mp. \| 342066 \| \| 6 \| late.mp. \| 485524 \| \| 7 \| initiation.mp. \| 304064 \| \| 8 \| exp health care utilization/ or utilization.mp. \| 311668 \| \| 9 \| exp prenatal care/ or 'Delayed attendance'.mp. \| 131988 \| \| 10 \| exp patient attendance/ or Attendance.mp. \| 28661 \| \| 11 \| exp pregnancy/ or exp prenatal care/ or 'Late booking'.mp. or exp mother/ or exp prenatal diagnosis/ \| 859069 \| \| 12 \| exp prenatal care/ or exp pregnant woman/ or exp pregnancy/ or booking.mp. \| 795139 \| \| 13 \| 1 or 2 or 3 or 4 or 5 or 6 or 7 or 8 or 9 or 10 or 11 or 12 \| 2244549 \| \| 14 \| 'Antenatal care'.mp. or exp prenatal care/ \| 133994 \| \| 15 \| exp pregnancy/ or exp prenatal care/ or exp pregnant woman/ or 'First antenatal care'.mp. or exp pregnancy complication/ \| 828364 \| \| 16 \| 'Prenatal care'.mp. or exp prenatal care/ \| 134573 \| \| 17 \| exp preeclampsia/ or exp prenatal care/ or exp pregnancy/ or 'Antepartum care'.mp. or exp fetus monitoring/ or exp pregnancy complication/ \| 819790 \| \| 18 \| exp pregnancy/ or exp prenatal care/ or ANC.mp. or exp pregnant woman/ \| 798998 \| \| 19 \| PNC.mp. \| 1204 \| \| 20 \| exp prenatal care/ or exp follow up/ or exp pregnancy/ or 'Antenatal follow up'.mp. or exp prenatal diagnosis/ or exp gestational age/ \| 1962131 \| \| 21 \| 'Antenatal period'.mp. or exp prenatal period/ \| 9426 \| \| 22 \| exp pregnancy/ or exp maternal care/ or exp prenatal care/ or 'Antenatal service'.mp. or exp health service/ or exp pregnant woman/ \| 5144221 \| \| 23 \| exp health service/ or exp pregnancy/ or exp pregnant woman/ or exp prenatal care/ or 'Antenatal care visit'.mp. \| 5144217 \| \| 24 \| exp prenatal diagnosis/ or exp pregnancy/ or 'Antenatal presentation'.mp. \| 756474 \| \| 25 \| exp pregnant woman/ or exp pregnancy/ or exp prenatal care/ or exp health care facility/ or 'Antenatal attendance'.mp. \| 2147974 \| \| 26 \| 'Perinatal care'.mp. or exp perinatal care/ \| 50592 \| \| 27 \| 'Maternal health services'.mp. or exp maternal health service/ \| 891 \| \| 28 \| 14 or 15 or 16 or 17 or 18 or 19 or 20 or 21 or 22 or 23 or 24 or 25 or 26 or 27 \| 5936151 \| \| 29 \| exp prevalence/ or 'Associated factors'.mp. or exp cross-sectional study/ \| 731241 \| \| 30 \| 'Factors associated'.mp. \| 117675 \| \| 31 \| 'Influencing factors'.mp. \| 7930 \| \| 32 \| Predictors.mp. or exp predictor variable/ \| 261656 \| \| 33 \| 'Predicting factors'.mp. \| 1581 \| \| 34 \| Correlates.mp. \| 182554 \| \| 35 \| Determinants.mp. or exp "social determinants of health"/ \| 154332 \| \| 36 \| 'Risk factors'.mp. or exp risk factor/ \| 962833 \| \| 37 \| 29 or 30 or 31 or 32 or 33 or 34 or 35 or 36 \| 2073703 \| \| 38 \| Ethiopia.mp. or exp Ethiopia/ \| 12770 \| \| 39 \| 13 and 28 and 37 and 38 \| 826 \| \| 40 \| limit 39 to (english language and yr="2002 - 2017") \| **778** \| | |
| **4** | **CINAHL** | \| 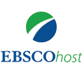 \|  \| \| --- \| --- \|  \| **#** \| **Query** \| **Limiters/Expanders** \| **Last Run Via** \| **Results** \| \| --- \| --- \| --- \| --- \| --- \| \| S8 \| S1 AND S4 AND S5 AND S6 \| Limiters - Published Date: 2002-01-01-2017-04-30; English Language  Search modes - Boolean/Phrase \| Interface - EBSCOhost Research Databases  Search Screen - Advanced Search  Database - CINAHL Complete \| **79** \| \| S7 \| S1 AND S4 AND S5 AND S6 \| Search modes - Boolean/Phrase \| Interface - EBSCOhost Research Databases  Search Screen - Advanced Search  Database - CINAHL Complete \| 79 \| \| S6 \| Ethiopia \| Search modes - Boolean/Phrase \| Interface - EBSCOhost Research Databases  Search Screen - Advanced Search  Database - CINAHL Complete \| 2,957 \| \| S5 \| Associated factors OR Factors associated OR Influencing factors OR Predictors OR Predicting factors OR ( Correlates or determinants ) OR ( Determinants or factors ) OR ( risk factors or protective factors ) \| Search modes - Boolean/Phrase \| Interface - EBSCOhost Research Databases  Search Screen - Advanced Search  Database - CINAHL Complete \| 1,124,069 \| \| S4 \| S2 OR S3 \| Search modes - Boolean/Phrase \| Interface - EBSCOhost Research Databases  Search Screen - Advanced Search  Database - CINAHL Complete \| 25,140 \| \| S3 \| Perinatal care OR Maternal health services \| Search modes - Boolean/Phrase \| Interface - EBSCOhost Research Databases  Search Screen - Advanced Search  Database - CINAHL Complete \| 10,468 \| \| S2 \| ( antenatal care or prenatal care ) OR First antenatal care OR Prenatal care OR Antepartum care OR ANC OR PNC OR Antenatal follow up OR Antenatal period OR Antenatal service OR Antenatal care visit OR Antenatal presentation OR Antenatal attendance \| Search modes - Boolean/Phrase \| Interface - EBSCOhost Research Databases  Search Screen - Advanced Search  Database - CINAHL Complete \| 16,121 \| \| S1 \| Delayed initiation OR Late initiation OR Delayed utilization OR late utilization OR Delayed OR late OR initiation OR utilization OR Delayed attendance OR Attendance OR Late booking OR Booking \| Search modes - Boolean/Phrase \| Interface - EBSCOhost Research Databases  Search Screen - Advanced Search  Database - CINAHL Complete \| 282,576 \| | |
